# Homology of homologous knotted proteins

**DOI:** 10.1098/rsif.2022.0727

**Published:** 2023-04-26

**Authors:** Katherine Benjamin, Lamisah Mukta, Gabriel Moryoussef, Christopher Uren, Heather A. Harrington, Ulrike Tillmann, Agnese Barbensi

**Affiliations:** ^1^ Mathematical Institute, University of Oxford, Oxford OX2 6GG, UK; ^2^ Wellcome Centre for Human Genetics, University of Oxford, Oxford OX3 7BN, UK; ^3^ Isaac Newton Institute for Mathematical Sciences, University of Cambridge, Cambridge CB3 0EH, UK; ^4^ School of Mathematics and Statistics, University of Melbourne, Melbourne, Victoria 3010, Australia

**Keywords:** persistent homology, knotted proteins, generators, topological statistical analysis

## Abstract

Quantification and classification of protein structures, such as knotted proteins, often requires noise-free and complete data. Here, we develop a mathematical pipeline that systematically analyses protein structures. We showcase this geometric framework on proteins forming open-ended trefoil knots, and we demonstrate that the mathematical tool, persistent homology, faithfully represents their structural homology. This topological pipeline identifies important geometric features of protein entanglement and clusters the space of trefoil proteins according to their depth. Persistence landscapes quantify the topological difference between a family of knotted and unknotted proteins in the same structural homology class. This difference is localized and interpreted geometrically with recent advancements in systematic computation of homology generators. The topological and geometric quantification we find is robust to noisy input data, which demonstrates the potential of this approach in contexts where standard knot theoretic tools fail.

## 1. Introduction

Since the first knotted and deeply knotted proteins were discovered [1–4], over 2000 knotted protein structures have been catalogued [5]. It is known that the knotted domains in families of proteins with significant sequence differences have been conserved throughout evolution [6].

Topological and geometric differences in knotted proteins correspond to differences in their folding pathways and dynamics as well as in their structural stability [5,7–13]. In particular, the location and relative length of the entanglement (known as *knot depth*) significantly influences the folding behaviour of trefoil proteins [10]. Perhaps most interesting is the case of knotted and unknotted carbamoyltransferases (AOTCases and OTCases), a pair of homologous proteins (i.e. proteins with strong sequence and structural similarities), where a single crossing change which creates the entanglement is responsible for an increased stability of the knotted structures, as shown by simulations [14]. To fully understand the interplay among entanglement, function and folding, it is necessary to incorporate a nuanced topological and geometric characterization of protein structure alongside sequence and functional analysis.

Recent studies have approached the characterization of knotted protein shape with techniques arising from low-dimensional topology and knot theory [9,15,16], resulting in significant improvements in the classification of open knots and a better understanding of the knot folding mechanism [13]. Although they are very powerful, low-dimensional topological techniques are computationally expensive and require precise and accurate input data.

Persistent homology (PH), the predominant tool in computational topology [17–19], has enabled the characterization of meaningful topological and geometric features in data. PH quantifies features such as loops and voids in data at multiple scales of resolution, which can be taken as a fingerprint of the shape of data. Advances in PH, in both computational speed [20] and statistical tools [21,22], have enabled the analysis of complex real-world datasets [23–26], including protein structure and folding [27–30]. A direct interpretation of homology features requires computing specific homology generators; their automatic and efficient computation is only recently possible [31].

In this work, we combine PH and low-dimensional topology to characterize geometric features of (open) knotted proteins. To our knowledge, the only other work linking PH and low-dimensional topology is new and considers only closed knots [32]. In a different direction, Mapper [33], another tool from computational topology, has been applied to the abstract collection of closed knots [34] parametrized by the values taken by polynomial invariants rather than the geometry of any specific embeddings. We propose and implement a computationally feasible PH pipeline for studying knotted proteins, at a global (full sequence) and local (substructural) scale, which does not rely on complex and computationally expensive knot invariants and sub-chain analysis [5,9,13,16]. Briefly, we input the three-dimensional coordinates of a knotted protein’s structure, construct a point cloud spanning the backbone of the protein, compute the corresponding PH groups and then translate these to persistence landscapes for protein comparison. We show that the information encoded in persistence diagrams and landscapes is enough to recover a clustering of the set of trefoil-knotted proteins by sequence similarity, thus validating PH as an effective fingerprint of protein structure. With this PH pipeline, we distinguish deeply versus shallowly knotted trefoil proteins and quantify structural differences between similar protein sequence homology classes. Computation of homology generators [31] has been used to show that the most persistent loops in PH correspond to active sites in the protein [29]; here, we show further that it distinguishes homologous knotted and unknotted proteins.

In particular, we are able to isolate the single local change that creates non-trivial entanglement in knotted AOTCases. Topological statistics [21,22] allows us to demonstrate that these results are statistically significant. We showcase the robustness of this pipeline to incomplete or corrupt sequence data, which arises with many biopolymers [5,35,36]. Furthermore, the pipeline does not depend on any particular assumptions about the underlying molecular graph, making it compatible with other theories of protein entanglement [37–39]. Hitherto, such analysis of noisy entangled curves is out of reach with a knot theoretic analysis.

## 2. Overview of dataset and pipeline

### 2.1. Dataset

**Figure 1.**
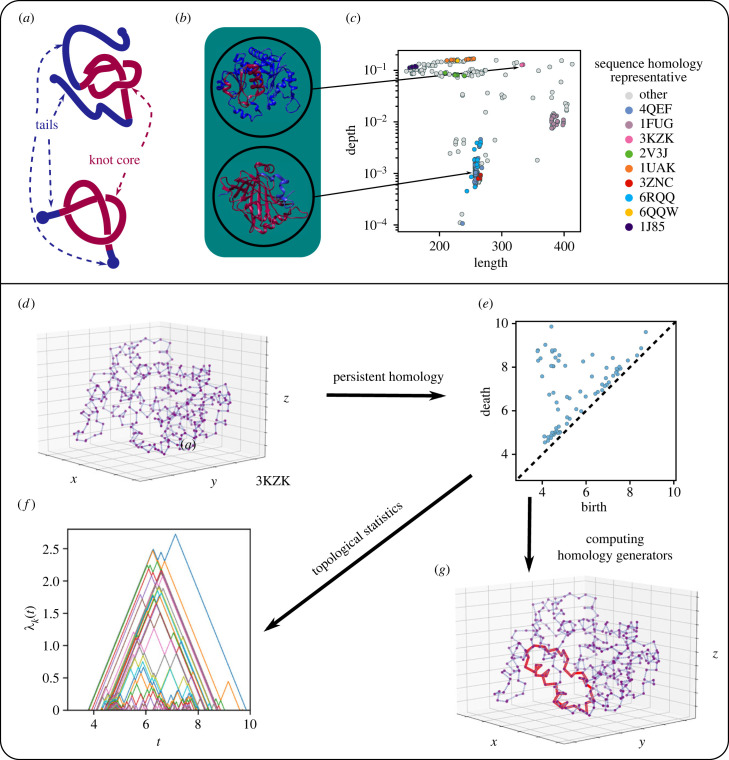
Dataset and PH pipeline. (Dataset) (*a*) Schematics of a deeply knotted (top) and shallowly knotted (bottom) open curve. Knot cores tails are shown in purple and blue. (*b*) Example of a deeply knotted protein (PDB entry 3KZK) and a shallowly knotted one (PDB entry 4QEF). (*c*) The space of trefoil-knotted proteins plotted by chain length and knot depth. Each protein is coloured according to its sequence homology class. Note that there are deeply and shallowly knotted proteins of the same length, as well as distinct sequence homology classes exhibiting similar length and depth. (Pipeline) (*d*) The protein dataset is given by lists of three-dimensional coordinates of C_*α*_ atoms. For each protein, we generate the point cloud consisting of these points and linearly interpolated points between each successive C_*α*_ atom. (*e*) Persistence diagram derived from the 3KZK point cloud. The points represent one-dimensional features corresponding to loops, and their positions represent the lifetimes of these features: their coordinates are their birth and death scales. (*f*) Persistence landscape derived from 3KZK. (*g*) PH generators in homology degree one can be represented by PL cycles whose vertices are points in the point cloud. In red, an example of a local generator for a one-dimensional feature of the 3KZK point cloud.

The first dataset we consider consists of proteins whose backbones form an open-ended positive trefoil knot [5]. These proteins form a large and well-studied part of all knotted proteins. The *knot core* of a protein is defined as the shortest knotted sub-chain in its backbone. A protein is *deeply knotted* if its knot core is entirely contained in a small portion of the chain, placed far away from the endpoints (figure 1*a*–*c*). Depth can be formally defined and quantified [9]. We label trefoil proteins as *shallow*, *deep* or *neither* based on their depth value. We also subdivide these trefoil-knotted proteins in the dataset into structural homology classes based on sequence similarity. Note that this division into classes can be performed using standard tools, see electronic supplementary material, section 1. The second dataset consists of unknotted proteins sharing the same structural homology class as 3KZK, a deeply knotted protein. This unknotted class is represented by 4JQO.

### 2.2. Pipeline

For a given protein, we first construct a point cloud approximating its backbone curve, figure 1*d*, and then compute the PH of this point cloud in homology degree one. The result of this computation is summarized in the persistence diagram, figure 1*e*, where each point with coordinates (*b*,*d*) represents a one-dimensional feature in the point cloud that is born at scale *b* and dies at scale *d*. Next we translate the persistence diagram into the corresponding collection of landscapes (figure 1*f*). A point (*b*,*d*) in the persistence diagram corresponds in the persistence landscape to a peak of height (*d* − *b*)/2 supported on the interval [*b*,*d*]. Persistence diagrams and persistence landscapes are two equivalent representations of PH. Next, for a given point (*b*,*d*) of the persistence diagram or peak in the persistence landscape, we find a homology generator. A generator is represented by a sequence of sub-chains of the protein backbone which are linked end-to-end to form a loop, called a *cycle* (figure 1*g*). This representative can be interpreted as a geometric localization of the topological feature encoded by the point in the persistence diagram. If desired, our pipeline presented here for backbone knotted proteins can be extended to all graphically presented proteins.

### 2.3. Experiments

For the first experiment, we consider two distinct measures of distance on PH: the Wasserstein distance on persistence diagrams and the *L*_1_ distance on persistence landscapes. Each distance induces a distinct dissimilarity measure on protein space, which we subsequently interpret using the dimensionality reduction algorithm Isomap [40]. We infer the typical PH of each structural homology class by computing its average persistence landscape.

For the second experiment, we consider a collection of trefoil-knotted and unknotted proteins belonging to the same structural homology class. We compute a generator of a one-dimensional feature which is unique to the PH of the knotted class.

In the third experiment, we reproduce the results of the first two experiments (i) with a sparser sampling of the protein backbone and (ii) after perturbing each point cloud by increasing amounts of Gaussian noise.

## 3. Results

### 3.1. Global clustering and depth type detection

**Figure 2.**
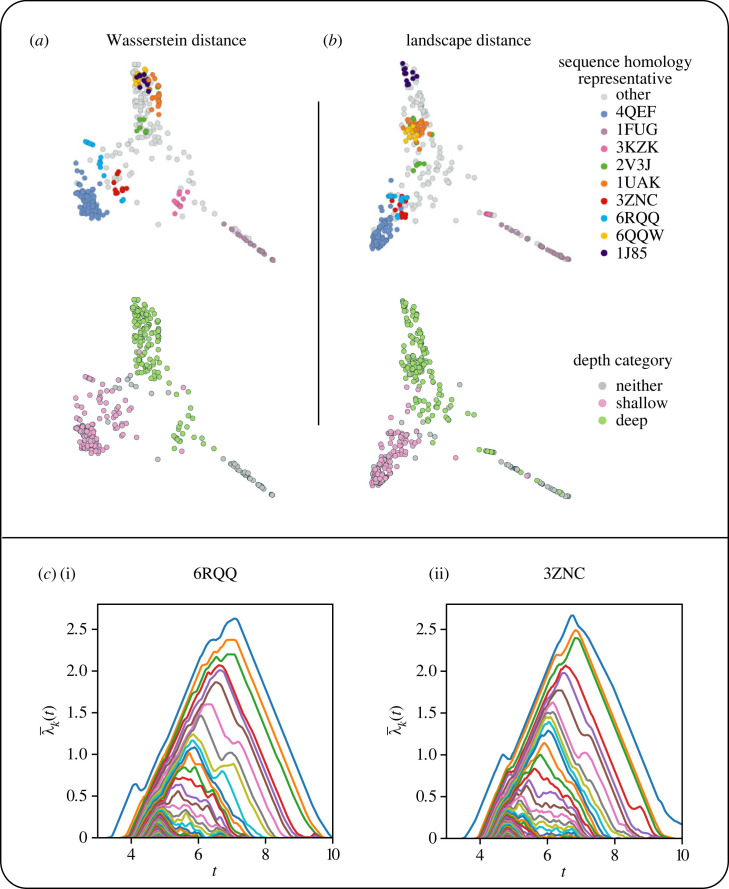
Global analysis: the space of knotted protein structures. (*a*) Isomap embedding of the space of trefoil-knotted proteins equipped with the Wasserstein distance on persistence diagrams (see electronic supplementary material, section 2). Given a distance matrix, isomap produces a configuration, two dimensional in our case, such that the new distance between any two objects is preserved as much as possible. The embedding forms clusters corresponding to sequence homology classes. The embedding successfully clusters by depth category. (*b*) Isomap embedding of the space of trefoil-knotted proteins equipped with the distance on persistence landscape. For a definition of this distance, see electronic supplementary material, section 2. The embedding forms clusters corresponding to sequence homology classes. The embedding successfully clusters by depth category. (*c*) Average persistence landscapes generated from the sequence homology classes with representative (i) 6RQQ and (ii) 3ZNC. Although these classes are not separated in the isomap embeddings in (*a*,*b*), a randomization test confirms that the difference in their average landscapes is statistically significant (*p* ≈ 0.003).

We demonstrate that PH captures structural, geometric and topological differences in proteins whose backbones form trefoil knots. Firstly, we compute the persistence diagrams and landscapes in dimension one for every protein in the dataset, and then pairwise distances between persistence diagrams and landscapes using the Wasserstein and *L*_1_ distances, respectively. The resulting structures induced by these distances on the space of trefoil proteins are approximated by dimensionality reduction, shown in figure 2*a*,*b*.

Proteins that are in the same homology class cluster together by both topological distances, validating that the PH pipeline recovers structural features of knotted proteins. We also observe separation between distinct homology classes. In particular, those sequence homology classes exhibiting significantly different chain lengths or depth types are very well separated, e.g. 1FUG and 3KZK. While some of the largest structural homology classes overlap in the projections, taking both the Wasserstein and *L*_1_ landscape metrics fully distinguishes the different sequence homology classes. We emphasize that the two distance matrices and corresponding Isomap projections are computed from the same initial data: the PH of each protein point cloud.

We apply topological statistics to infer the PH shape of structural homology classes and detect geometric differences between proteins in these classes. We define the topological fingerprint of each structural homology class to be the average landscape of the class (figure 2*c*). We employed a 1000-sample randomization test to compare these topological fingerprints (see electronic supplementary material, section 2 and table S1). The test produced an approximate *p*-value of 0.001 for all pairs of classes except 3ZNC and 6RQQ, which gave a *p*-value of 0.003. Therefore, PH detects statistically significant pairwise differences between all structural homology classes, including those not separated in the isomap configurations.

By using this topological pipeline, we next detect knot depth, which is a meaningful geometric feature intrinsic to protein entanglement. Figure 2*a*,*b* shows a considerable separation between shallowly and deeply knotted proteins. In particular, even deeply knotted proteins and shallowly knotted proteins having similar chain length as those in the 4QEF and 1UAK classes are clearly separated. This shows that, beyond separating proteins by structural similarity, PH can be successfully employed to analyse the geometric entanglement of open curves.

### 3.2. Detecting local topological changes in homologous proteins

**Figure 3.**
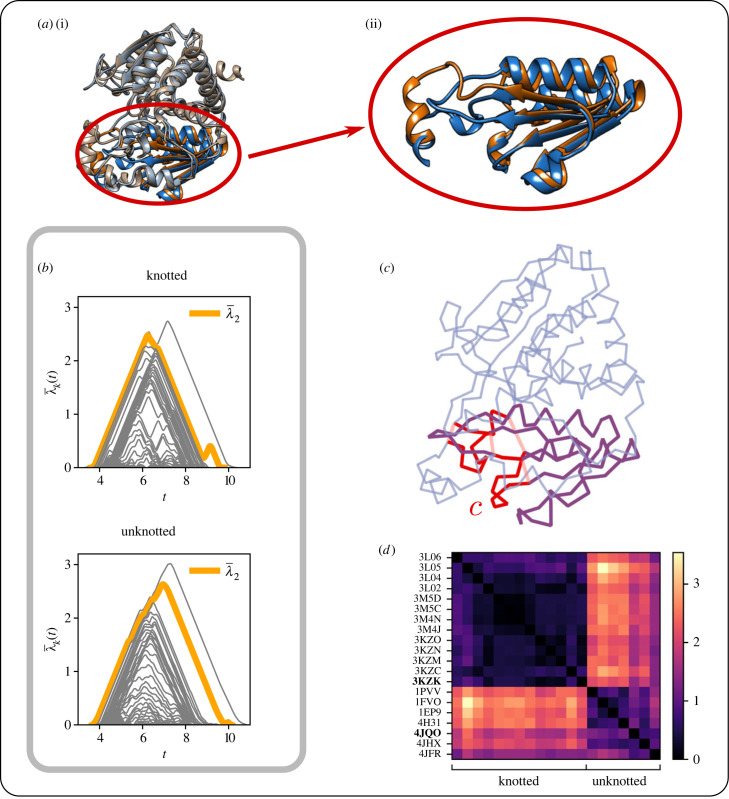
Local analysis: geometry of homologous protein substructures. (*a*) Two homologous proteins, 3KZK (blue, knotted) and 4JQO (orange, unknotted), overlaid. These proteins have almost superimposable structures, but differ as knots by a crossing change localized within the red ellipse. The knot core in 3KZK and its corresponding structure in 4JQO are highlighted by showing the remaining parts in lighter shades of orange and blue. A close-up of the local configurations causing the topological change is shown in (*a*)(ii). A strand movement transforms the deeply knotted 3KZK into the unknotted 4JQO. (*b*) Average persistence landscapes generated from knotted (top) and unknotted (bottom) protein chains. The peak in *λ*_2_ (orange) centred at *t* ≈ 9 in the knotted case corresponds to a generator *c* for the PH of the knotted chains which does not arise in the PH of the unknotted chains. (*c*) The backbone of 3KZK. Violet segments indicate the knot core. The cycle representing the PH generator *c* is plotted in red and pink, where the pink segments show simplices in *c* that are not part of the 3KZK curve. Note that *c* is positioned close to the knot core and, more specifically, close to the crossings responsible for the non-trivial entanglement. Further, *c* intersects the arc that needs to be pushed to untangle the curve. (*d*) Heat map showing the distances between the *λ*_2_ landscapes for the proteins in the AOTCase and OTCase families. The two distinct purple squares demonstrate sufficient similarity in each class for the average *λ*_2_ landscapes to be faithful representatives for each class.

We now investigate whether PH is sensitive to knottedness. We consider the homologous pair of protein structures: the trefoil-knotted 3KZK and the unknotted 4JQO. As shown in figure 3*a*, the difference in structure between these proteins is localized in a few separate non-overlaying portions. One of these portions encloses the crossing change responsible for the change in the knot type, and a natural question is whether PH is capable of detecting and localizing this topological difference. To this end, we form a new dataset consisting of all the trefoil-knotted AOTCases (of which 3KZK can be taken as a representative) and the unknotted OTCases (of which 4JQO can be taken as a representative) [7,41].

The average persistence landscapes for these two classes are displayed in figure 3*b*. Despite the high similarity of all the protein structures considered here, we notice remarkable differences between the two average landscapes, most notably in the *λ*_2_ landscape layers (figure 3*b*, orange curve).

For the trefoil-knotted AOTCase family, *λ*_2_ has a small second peak centred at scale value 9, which is not present for the unknotted OTCase family. To investigate this difference, we compare only the *λ*_2_ layer for each of the proteins in the two classes. The heat map shown in figure 3*d* confirms that the *λ*_2_ layers within each of the AOTCase and OTCase families are sufficiently similar to each other; thus, the average *λ*_2_ landscape layer is a good representative of each class.

We geometrically interpret the *λ*_2_ layer difference by analysing the corresponding homology generators. We focus on the *λ*_2_ layers of specific proteins, namely, the knotted AOTCase 3KZK and the OTCase 4JQO. In the landscape of 3KZK, the *λ*_2_ peak corresponds to a specific PH generator *c* via the landscape diagrams correspondence [21]. A cycle representing the PH generator *c* was computed using a recently available and computationally efficient algorithm in Eirene [31] (figure 3*c*, red). Most of the simplices forming the cycle are part of the knot core (figure 3*c*, violet) of the protein backbone. The homology generator identifies the portion of the protein backbone that forms the protein knot. Specifically, the cycle overlaps with the essential crossing that distinguishes the knotted and unknotted proteins. We compute and find a similar result across all of the AOTC structures. Note that, for a given homology class, homology generators are not unique, and in general, different representatives might differ substantially [42]. However, recent empirical results on spatial curves show that homology generators in degree one output by the Eirene software do not differ greatly from canonical minimal generators in this special case [43]. Indeed, in our work, we find that the same representative is repeatedly localized across all the AOTC structures, including when the curve is perturbed by noise as discussed in the following section.

### 3.3. The persistent homology pipeline is robust

One of the main strengths of PH is its theoretically guaranteed robustness to noise [44]. A natural question is whether the results stand when the input data is incomplete or noisy. To check this, we first (i) apply the pipeline to a sparse point cloud, given directly by the C_*α*_ atoms for each protein—i.e. without interpolating between adjacent atoms. Note that our pipeline simplified in this way is agnostic to sequence and bonds. But despite the fact that this information is forgotten in the carbon atom point cloud, our PH analysis is able to recover precisely the same geometric and topological information, in particular knot depth and local changes in entanglement. We then (ii) perturb each point cloud by applying increasing amounts of Gaussian noise to the coordinates of each point and repeat the procedure yet again (see electronic supplementary material, section 4). Remarkably, we can successfully reproduce both the global and local results of the previous sections also in this case. The sequence homology clustering only fails when perturbing the points with a standard deviation of approximately one third the distance between two consecutive amino acids, while the crossing change detection is lost slightly earlier. Isomap plots and the corresponding local generator analysis are described in detail in electronic supplementary material, section 3 and figures S1 and S2.

## 4. Discussion

Our investigation illustrates the potential of using PH to analyse biopolymers with complex geometry. We showed Wasserstein distance and landscape distance meaningfully discriminated between protein structures without the computationally expensive pre-processing, such as sequence alignment, required for traditional methods.

While structural classification of proteins via PH has previously been explored in other works [27–30], only two have used the backbone curve as input data [27,30], and only the developer of landscapes used the statistical apparatus of persistence landscapes for this task [29]. We successfully employed persistence landscapes to distinguish between structurally similar knotted and unknotted proteins, thus detecting their global topology. Furthermore, we used generators to localize the differences between the topological types of homologous proteins. This proposed methodology has the potential to be applicable in other contexts in which knots arise naturally.

Here, we built, combined and integrated on all of these techniques to explore the space of knotted proteins, to compare their structure without requiring alignment and to detect features specific to open knots, e.g. knot depth. In addition to recovering structural similarity, our results demonstrate that PH contains coarse information on the geometric type of open knots. Furthermore, we demonstrated that our approach works with noisy data and could therefore be a computationally efficient tool for the study of knotted structures in other contexts. Our approach complements, rather than substitutes, the established knot-theoretical approaches [5,13,16] and offers an alternative to entanglement characterizations when the available data are imprecise or poorly resolved. In these cases, an analytical approach is infeasible and a coarse description of the entanglement type is more appropriate. Indeed, the robustness of our methodology could be particularly crucial in cases in which experiments are performed at a resolution that does not guarantee a complete determination of a biopolymer’s underlying spatial curve, as in the case of DNA chromosomes [35,36].

## 5. Material and methods

### 5.1. Protein data

We model each protein structure as the piecewise linear (PL) curve spanned by its C_*α*_ atoms, and we consider proteins whose backbones form an open-ended positive trefoil [5]. We construct a point cloud from its PL backbone curve by linearly interpolating a total of five equidistant points between each pair of consecutive C_*α*_ atoms in the curve. For more details, see electronic supplementary material, section 1.

### 5.2. Persistent homology computations

Persistence diagram in dimension one are computed using Ripser [45]. Pairwise *W*_1_[*L*_∞_]-Wasserstein distances are computed using GUDHI [46]. PH generators are computed using Eirene [31]. Persistence landscapes are computed using Python software, available at https://gitlab.com/kfbenjamin/pysistence-landscapes. Our software is based on the algorithms given in [22]. The software includes scripts for finding and plotting landscapes from a barcode, computing average landscapes and calculating distance. For more details, see electronic supplementary material, sections 2 and 4.

### 5.3. Noisy data

To probe our methodology’s robustness, we apply our pipeline to the non-interpolated point clouds obtained by considering only the C_*α*_ atoms and to successive perturbations of such point clouds. For more details, see electronic supplementary material, sections 2 and 4 and figures S1 and S2.

## Data Availability

The data and code for this work are available at https://github.com/katherine-benjamin/ph-knotted-proteins and archived at Zenodo: https://doi.org/10.5281/zenodo.7799281 [47]. The data are provided in electronic supplementary material [48].
